# Identity recognition in response to different levels of genetic relatedness in commercial soya bean

**DOI:** 10.1098/rsos.160879

**Published:** 2017-01-11

**Authors:** Guillermo P. Murphy, Rene Van Acker, Istvan Rajcan, Clarence J. Swanton

**Affiliations:** Department of Plant Agriculture, University of Guelph, 50 Stone Road East, Guelph, Ontario, CanadaN1G 2W1

**Keywords:** kin selection, identity recognition, competition, phenotypic plasticity, biomass allocation, genetic relatedness

## Abstract

Identity recognition systems allow plants to tailor competitive phenotypes in response to the genetic relatedness of neighbours. There is limited evidence for the existence of recognition systems in crop species and whether they operate at a level that would allow for identification of different degrees of relatedness. Here, we test the responses of commercial soya bean cultivars to neighbours of varying genetic relatedness consisting of other commercial cultivars (intraspecific), its wild progenitor *Glycine soja*, and another leguminous species *Phaseolus vulgaris* (interspecific). We found, for the first time to our knowledge, that a commercial soya bean cultivar, OAC Wallace, showed identity recognition responses to neighbours at different levels of genetic relatedness. OAC Wallace showed no response when grown with other commercial soya bean cultivars (intra-specific neighbours), showed increased allocation to leaves compared with stems with wild soya beans (highly related wild progenitor species), and increased allocation to leaves compared with stems and roots with white beans (interspecific neighbours). Wild soya bean also responded to identity recognition but these responses involved changes in biomass allocation towards stems instead of leaves suggesting that identity recognition responses are species-specific and consistent with the ecology of the species. In conclusion, elucidating identity recognition in crops may provide further knowledge into mechanisms of crop competition and the relationship between crop density and yield.

## 1. Introduction

Competition is one of the key processes that dominate plant–plant interactions [1–4]. Most plants display some degree of plasticity in the production of phenotypes involved in maximizing their competitive ability [5]. This phenotypic plasticity to competition in plants can be considered analogous to competitive behaviour in animals and is, among other factors, highly dependent on the degree of genetic relatedness of neighbours [6–9]. The more related two individuals are, the less they will benefit from producing competitive phenotypes that can harm their neighbours because they share genetic material. Kin selection theory states that any trait or behaviour that benefits a relative will be selected as long as the benefit to the relative is greater than the cost to the organism displaying the trait or behaviour [10].

Behaviours that benefit neighbours are stable from an evolutionary perspective only if specifically directed towards related rather than unrelated individuals [11]. For this, plants have evolved identity recognition systems that allow them to distinguish related from unrelated neighbours (reviewed by [7]). Identity recognition allows plants to recognize their competitors and tailor their responses accordingly [12]. Moreover, the ability of plants to respond plastically to the identity of neighbours has been proposed as a mechanism of intraspecific competition that allows plants to pre-empt and optimize competitive responses, i.e. kin recognition [7,12,13]. Species-specific kin recognition has been observed in several plant species with responses ranging from differences in biomass allocation [14–17], morphology [15,18–21] and growth [22]. In addition, kin recognition has also been shown to enhance symbiotic interactions with microbial partners [23] and defences to herbivory [24–25]. Most evidence for a mechanism of kin recognition suggests root exudates as a signal of relatedness [18,19,26] although aboveground volatiles [24] and light signals ([20] but see [27,28]) have also been proposed.

As a body of literature, identity recognition studies have focused on naturally occurring species as well as *Arabidopsis*. Very little is known, however, about identity recognition in crop species. Two studies looked at root placement behaviour in rice, soya bean and maize in response to growing with neighbours of the same or different cultivars [29,30]. They showed that more related plants tended to aggregate roots while less related plants grew roots away from each other. A different study, in turn, observed changes in root allocation in response to more related neighbours but only in a modern wheat cultivar and not in an older one, concluding that identity recognition may have evolved, or have been bred into, modern wheat cultivars [31]. While interesting, these studies only provide a first glance at the role of identity recognition in crops species.

Elucidating the role of identity recognition in crop species is important. While kin recognition influences intra-specific competition, species recognition influences interspecific interactions. Crop species are grown in highly managed environments under intensive weed management regimes and at densities of very highly related plants required to optimize yield. These growing conditions may make interactions with siblings more predictable for crops than for many wild plants. As a result, selection pressures for traits that benefit neighbours may be higher in crop species than in natural species. In theory, a crop cultivar that always invests the least possible energy in producing competitive traits in order to invest that energy into reproduction is predicted to have a higher yield than a cultivar that is more competitive [32]. This strategy is successful only as long as the crop is kept weed free. If weeds invade a crop, then cultivars that have identity recognition systems are predicted to be more successful, as these plants are able to identify their neighbours and respond less competitively towards more related neighbours. Thus, identity recognition may play an important role in the determination of weed removal strategies as well as optimal planting patterns and density for successful crop production. The existence of recognition systems in current cultivars of different commercial crops, however, cannot be assumed and needs to be tested empirically.

In this study, we investigated responses of commercial soya bean cultivars to the identity of neighbours with varying levels of genetic relatedness including different soya bean cultivars (*Glycine max* (L.) Merrill), the closest ancestral wild relative soya bean species *G. soja* Siebold & Zuccarini [33,34], and the unrelated leguminous species white bean (*Phaseolus vulgaris* L. cv. Bolt). We also report the reciprocal responses of the wild soya bean ancestral species to the commercial soya bean neighbours to elucidate differences among highly related species with different ecologies. We hypothesize that if commercial soya bean cultivars are capable of recognizing the identity of neighbours, then, based on kin selection theory, they will produce more competitive phenotypes in the presence of less related neighbours.

## 2. Material and methods

This study consisted of four experiments that tested soya bean (*G. max* (L.) Merr.) responses to neighbour identity at different levels of genetic relatedness assuming a mechanism of identity recognition mediated by root exudates [18,19,26]. Before planting, seeds of each species were pre-germinated on moist germination paper for 48 h. Germinated seeds containing an emerged root radicle were then planted into pots (14 × 19 cm; W × H) containing Turface MVP^®^ (Profile Products LLC, Buffalo Grove, IL, USA) as the soil media at a uniform depth of 2 cm. Turface is a fairly inert, loose clay-based soil media that was used to facilitate root extraction and to minimize any microbial interactions. In addition, no rhizobium species were added to the Turface mixture. Seeds were planted 4 cm apart and 2 cm from the centre of the pot. Plants were then placed in controlled environment growth chambers (Model PGW 36 Conviron or Model GRC-36 Canada Bio Chambers, Winnipeg, Manitoba, Canada) at 26°C (daytime) and 19°C (night time) with a 16 h photoperiod. Photosynthetic photon flux density was 700 µmol m^−2 ^s^−1^ and relative humidity was 65%. R:Fr from incoming radiation was measured at 3.5 µmol m^−2^ s^−1^ while R:Fr from reflected radiation of the neighbouring plants was 3.0 µmol m^−2^ s^−1^ from wild soya bean, 2.8 µmol m^−2^ s^−1^ from commercial soya bean, and 2.7 µmol m^−2^ s^−1^ from white beans at time of harvest. Pots were placed randomly within the chamber and re-randomized for every replicate to minimize within chamber effects. Plants were fertilized weekly with 20-20-20 NPK fertilizer (Plant-Prod fertilizer, Plant Production Co., Brampton, Ontario, Canada).

### 2.1. Experiment 1: intra-specific recognition

**Figure 1.**
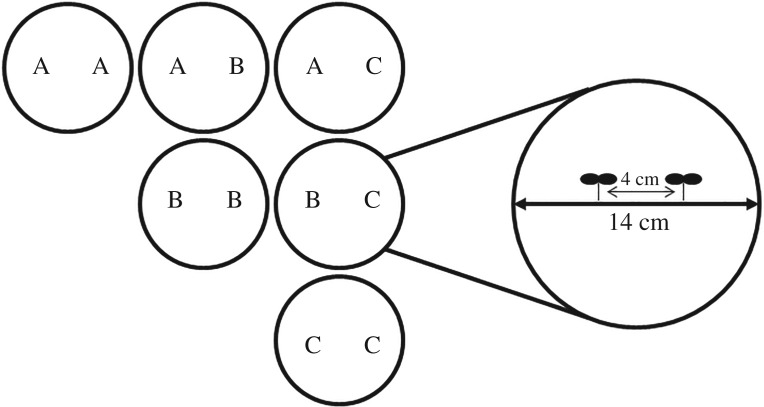
Two-dimensional representation of a typical pot from a top view and six pots depicting all possible combinations of cultivar interactions. A, B and C refer to soya bean cultivars OAC Wallace, OAC Calypso and OAC Champion, respectively. Pots with two plants of the same letter represent the same cultivar (SC) treatment while pots with two plants of different letters represent the different cultivar (DC) treatment. Distance between plants was 4 cm and pot diameter was 14 cm.

This experiment compared morphological and behavioural responses of three different University of Guelph commercial soya bean cultivars (OAC Wallace, OAC Calypso and OAC Champion) to the identity of the neighbouring soya bean cultivar. In this group, OAC Wallace and OAC Champion were related as they shared one parent in common whereas both were completely unrelated to OAC Calypso (I. Rajcan 2016, personal communication). The experiment included all possible neighbour cultivar combinations creating a relatedness treatment consisting of the same cultivar (SC) or a different cultivar (DC) (figure 1). We also compared the same traits among cultivars independent of the relatedness treatment as well as interactions between relatedness and cultivars. The experiment consisted of four replicates. Each replicate comprised 12 pots of each possible neighbour cultivar combination for a sample size of 24 plants for each treatment combination per replicate, resulting in a final sample size of *N* = 576. All plants were harvested at the V3 (third trifoliate leaf fully expanded) stage of soya bean development [35].

### 2.2. Experiments 2: related species recognition

Two experiments were conducted to explore morphological and behavioural responses to the identity of a neighbour belonging to a highly related species. In the first experiment, we measured how the commercial cultivar (OAC Wallace) responded to the identity of wild soya bean (*G. soja*, Siebold & Zucc), its ancestral species [33,34]. The second experiment measured how the wild soya bean responded to the same commercial cultivar. Both experiments used a pairwise design (following the methodology of Bhatt *et al.* [16]). Both experiments were replicated five times. In each replicate, both plants were measured in pots of the same species treatment while only one plant (species of interest) was measured in pots containing different species. As a result, each replicate comprised 12 pots of the same species treatment and 24 pots of the different species treatment resulting in a sample size of 24 plants per treatment combination per replicate, and a final sample size of *N* = 240. All plants were harvested at the V2 (second trifoliate leaf fully expanded) stage of soya bean development.

### 2.3. Experiment 3: unrelated species recognition

This experiment compared morphological and behavioural responses of a University of Guelph commercial soya bean cultivar (OAC Wallace) to the identity of neighbouring plants of an unrelated species (white bean, *P. vulgaris*, L. cv. Bolt). The experimental design was identical to that described in Experiment 2. The experiment was replicated five times. This design allowed for a sample size of 24 plants per treatment combination per replicate, and a final sample size of *N* = 240. Harvesting occurred at the V3 stage of soya bean development.

### 2.4. Data collection

Prior to harvest, all plants were tagged at the soil surface in order to maintain correct identification of roots and shoots. At each harvest time, the aboveground portions of the plants were clipped manually at the soil surface and partitioned into leaves and stems. The remaining root systems were then hand-washed, separated carefully into individual roots and matched to their corresponding aboveground shoots. Leaves, stems and roots for each individual plant were bagged separately and dried at 60°C to a constant weight. Once the plant material was dried, internode length for each stem was measured and all plant components were weighed separately to obtain biomass measurements.

### 2.5. Data analysis

The data were analysed using SAS statistical software (v. 9.3; SAS Institute, Cary, NC, USA). PROC MIXED was used to carry out analyses of variance and covariance. Analysis of covariance was used to test for differences in allocation and stem elongation [36,37]. Relatedness treatments were declared as fixed effects while replication and replication by treatment interactions were declared as random effects. All replications occurred over time. Stem elongation was measured as the least square mean from an analysis of covariance with plant total height (summation of recorded internode length per plant) as the dependent variable and stem biomass as the covariate (LSMEANS option, PROC MIXED). Differences in the estimated relation between components of plant biomass rather than proportional biomass ratios were used to test for differences in allocation [36,38]. Allocation to roots was measured as the least square means from an analysis of covariance with root biomass as the dependent variable and stem biomass as the covariate. For allocation to leaves, leaf biomass was used as the dependent variable and stem biomass as the covariate. Root to leaf allocation was log-transformed to ensure that the residual variance was homoscedastic, and the distribution of the residuals did not differ significantly from normality. Parameters are presented untransformed for clarity. Data with *p*-values slightly above 0.05 (0.05 < *p* < 0.06) were still considered significant.

## 3. Results

No significant differences were detected in the traits measured when commercial soya bean cultivars were grown with the SC or in combination with any other soya bean cultivar. Despite the lack of differences to relatedness in intraspecific interactions, all three cultivars showed intrinsic differences between each other in terms of stem elongation (*p* = 0.0021) and total plant biomass (*p* = 0.0513) (table 1). OAC Champion had the most elongated stem (elongation = 9.54 cm) followed by OAC Wallace (elongation = 8.85 cm) and OAC Calypso (elongation = 8.48 cm) (*p* = 0.0021). Total plant biomass of OAC Champion differed from OAC Calypso (OAC Champion plant biomass = 0.92 g, OAC Calypso plant biomass = 0.84 g, *p* = 0.0193). OAC Wallace, however, had an intermediate plant biomass of 0.88 g that did not differ from OAC Champion (*p* = 0.2113) or OAC Calypso (*p* = 0.1172).

**Table 1.** Analyses of variance and covariance for five traits of commercial soya bean (*G. max*) in response to intra-specific neighbour relatedness and cultivar identity across relatedness treatments. Stem biomass was used as covariate for elongation, root/stem and leaf/stem allocation. Leaf biomass was used as covariate for root/leaf allocation. Italics indicate significant values.

		total biomass		elongation		root/stem allocation		root/leaf allocation		leaf/stem allocation	
covariance parameters		*Z*	*PrZ*	*Z*	*PrZ*	*Z*	*PrZ*	*Z*	*PrZ*	*Z*	*PrZ*
replicate		1.2	0.1124	1.1	0.1270	1.2	0.1129	1.2	0.1120	1.2	0.1221
Rep × Rel		0.4	0.3341	—	—	0.2	0.4093	0.3	0.3653	0.8	0.2121
Rep × Cul		—	—	0.6	0.2763	1.1	0.1362	1.1	0.1330	0.2	0.4094
residual		15.8	*<0*.*0001*	15.7	*<0*.*0001*	15.7	*<0*.*0001*	15.7	*<0*.*0001*	15.7	*<0*.*0001*

fixed effects	ndf/ddf	*F*	*p*	*F*	*p*	*F*	*p*	*F*	*p*	*F*	*p*
relatedness	1/3	0.02	0.8996	0.7	0.4580	0.2	0.6616	0.2	0.6858	0.9	0.4125
cultivar	2/6	5.08	*0*.*0513*	20.4	*0*.*0021*	3.6	0.0930	2.6	0.1545	2.0	0.2228
Rel × Cul	2/491	1.18	0.3077	0.1	0.9519	0.5	0.6132	0.01	0.9856	1.6	0.1982
covariate	1/491	—	—	301.4	*<0*.*0001*	765.8	*<0*.*0001*	1093.8	*<0*.*0001*	1765.2	*<0*.*0001*

**Figure 2.**
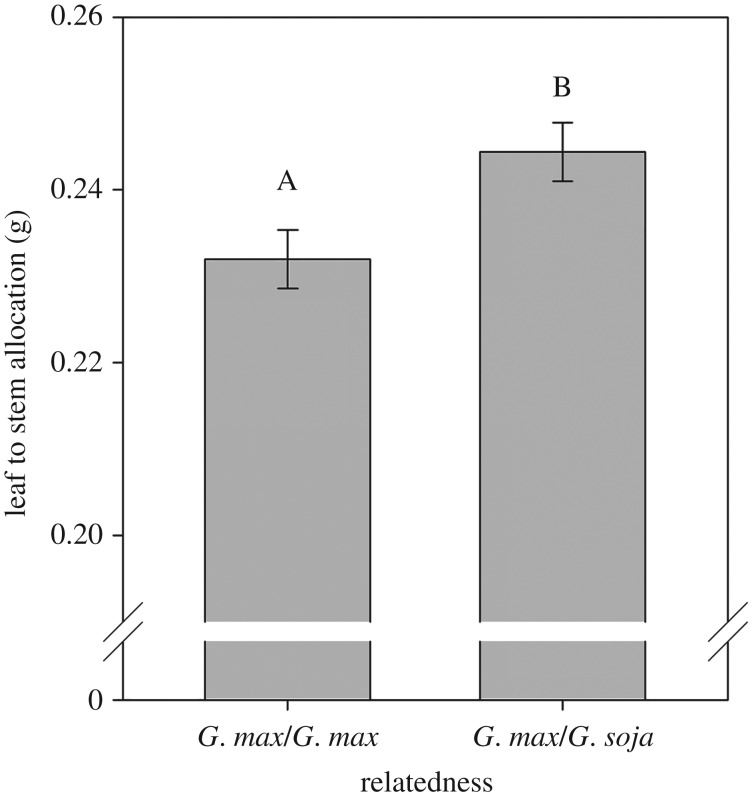
Effects of neighbour relatedness (a neighbour of the same cultivar or a neighbour of *G. soja*) on leaf allocation of commercial soya bean (*G. max* var. OAC Wallace). Letters at the top of the bars represent significant differences at the 0.05 level. Bars indicate 1 s.e.

Significant differences to the identity of neighbours were detected among related species. The commercial cultivar OAC Wallace increased allocation to leaves compared with stems (figure 2) and increased total plant biomass when growing with neighbours of its ancestral species, wild soya bean (with commercial soya bean neighbour = 0.47 g, with wild soya bean neighbour = 0.50 g, s.e. = 0.01, *p* = 0.0567). No differences were observed in elongation, root to stem or root to leaf allocation (table 2).

**Table 2.** Analyses of variance and covariance for five traits of commercial soya bean (*G. max*) in response to neighbour relatedness (same cultivar or *G. soja*). Stem biomass was used as covariate for elongation, root/stem and leaf/stem allocation. Leaf biomass was used as covariate for root/leaf allocation. Italics indicate significant values.

		total biomass		elongation		root/stem allocation		root/leaf allocation		leaf/stem allocation	
covariance parameters		*Z*	*PrZ*	*Z*	*PrZ*	*Z*	*PrZ*	*Z*	*PrZ*	*Z*	*PrZ*
replicate		1.6	0.0601	1.4	0.0796	1.5	0.0615	1.5	0.0692	1.5	0.0620
Rep × Rel		—	—	0.8	0.2244	—	—	—	—	—	—
residual		10.5	*<0*.*0001*	10.4	*<0*.*0001*	10.5	*<0*.*0001*	10.5	*<0*.*0001*	10.5	*<0*.*0001*

fixed effects	ndf/ddf	*F*	*p*	*F*	*p*	*F*	*p*	*F*	*p*	*F*	*p*
relatedness	1/5	6.09	*0*.*0567*	0.5	0.5334	4.8	0.0792	0.7	0.4487	13.7	*0*.*0141*
covariate	1/214	—	—	263.7	*<0*.*0001*	399.6	*<0*.*0001*	734.2	*<0*.*0001*	495.8	*<0*.*0001*

**Figure 3.**
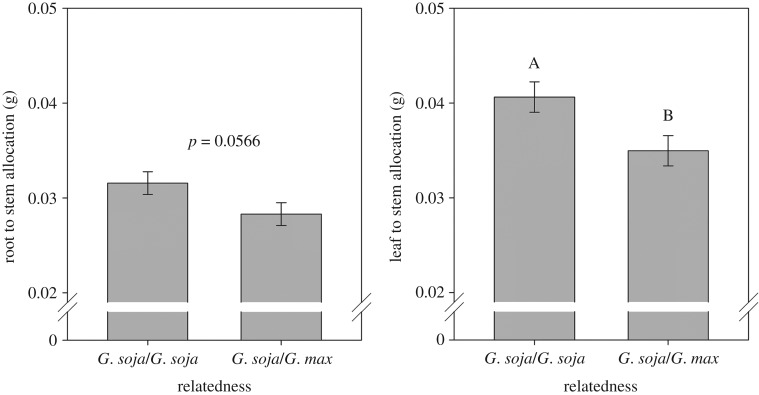
Effects of neighbour relatedness (a neighbour of the same cultivar or a neighbour of *G. max*) on root to stem allocation and leaf to stem allocation of wild soya bean (*G. soja*). Letters at the top of the bars represent significant differences at the 0.05 level. Bars indicate 1 s.e.

Wild soya bean plants also showed significant differences to the identity of neighbours of the related commercial soya bean. Wild soya bean plants decreased root to stem and leaf to stem allocation when growing with neighbours of OAC Wallace (figure 3). This decrease in leaf to stem allocation observed in wild soya bean was opposite to the response observed in OAC Wallace. Total biomass, stem elongation and root to leaf allocation, however, showed no response to neighbour identity in wild soya bean (table 3). Total biomass of wild soya bean, however, was approximately 17% that of commercial soya bean across treatments (0.08 g wild soya bean versus 0.48 g commercial soya bean), indicating a constitutive size difference among these species.

**Table 3.** Analyses of variance and covariance for five traits of wild soya bean (*G. soja*) in response to neighbour relatedness (same cultivar or *G. max*). Stem biomass was used as covariate for elongation, root/stem and leaf/stem allocation. Leaf biomass was used as covariate for root/leaf allocation. Italics indicate significant values.

		total biomass		elongation		root/stem allocation		root/leaf allocation		leaf/stem allocation	
covariance parameters		*Z*	*PrZ*	*Z*	*PrZ*	*Z*	*PrZ*	*Z*	*PrZ*	*Z*	*PrZ*
replicate		1.3	0.0923	1.2	0.1146	1.2	0.1092	1.2	0.1088	0.4	0.3585
Rep × Rel		0.4	0.3386	1.0	0.1562	0.7	0.2329	0.7	0.2483	1.0	0.1629
residual		8.4	*<0*.*0001*	8.4	*<0*.*0001*	8.4	*<0*.*0001*	8.4	*<0*.*0001*	8.4	*<0*.*0001*

fixed effects	ndf/ddf	*F*	*p*	*F*	*p*	*F*	*p*	*F*	*p*	*F*	*p*
relatedness	1/14	0.98	0.3783	0.1	0.8195	7.1	*0*.*0566*	0.4	0.5858	11.77	*0*.*0265*
covariate	1/140	—	—	373.7	*<0*.*0001*	272.3	*<0*.*0001*	729.9	*<0*.*0001*	480.88	*<0*.*0001*

**Figure 4.**
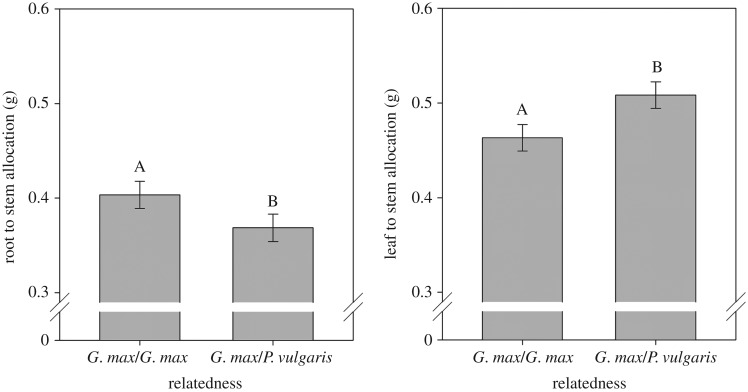
Effects of neighbour relatedness (a neighbour of the same cultivar or a neighbour of *P. vulgaris*) on root to leaf allocation, and leaf to stem allocation for commercial soya bean (*G. max* var. OAC Wallace). Letters at the top of the bars represent significant differences at the 0.05 level. Bars indicate 1 s.e.

OAC Wallace also showed recognition to the identity of neighbours of an unrelated species. In response to growing with neighbouring plants of *P. vulgaris* cv. Bolt, OAC Wallace allocated less to roots compared with leaves (figure 4*a*) and more to leaves compared with stem (figure 4*b*). The observed increase in leaf to stem allocation was consistent with the response observed from OAC Wallace to neighbours of wild soya bean described above. The decrease in root to leaf allocation, however, occurred only with the unrelated species *P. vulgaris*. There were no differences in total biomass, stem elongation or root to stem allocation in response to neighbour identity of *P. vulgaris* (table 4).

**Table 4.** Analyses of variance and covariance for five traits of commercial soya bean (*G. max*) in response to neighbour relatedness (same cultivar or *P. vulgaris*). Stem biomass was used as covariate for elongation, root/stem and leaf/stem allocation. Leaf biomass was used as covariate for root/leaf allocation. Italics indicate significant values.

		total biomass		elongation		root/stem allocation		root/leaf allocation		leaf/stem allocation	
covariance parameters		*Z*	*PrZ*	*Z*	*PrZ*	*Z*	*PrZ*	*Z*	*PrZ*	*Z*	*PrZ*
replicate		1.2	0.1132	1.1	0.1377	1.3	0.0975	1.4	0.0801	1.3	0.0899
Rep × Rel		1.3	0.1010	1.2	0.1242	0.8	0.2020	—	—	1.0	0.1619
residual		10.5	*<0*.*0001*	10.5	*<0*.*0001*	10.4	*<0*.*0001*	10.5	*<0*.*0001*	10.5	*<0*.*0001*

fixed effects	ndf/ddf	*F*	*p*	*F*	*p*	*F*	*p*	*F*	*p*	*F*	*p*
relatedness	1/4	4.2	0.1105	438.3	0.2859	0.5	0.5313	38.6	*0*.*0034*	10.77	*0*.*0305*
covariate	1/220	—	—	1.5	*<0*.*0001*	535.6	*<0*.*0001*	1405.8	*<0*.*0001*	715.36	*<0*.*0001*

## 4. Discussion

In this study, we examined the responses of commercial soya bean plants to the presence of neighbours of varying genetic relatedness. The main goal was to elucidate whether commercial soya bean plants were capable of identity recognition, an ability identified in several wild plant species (as reviewed by [7]). We also investigated responses of wild soya bean to serve as a comparison with its commercial counterpart. Testing the responses of commercial soya bean plants to neighbours of different genetic relatedness allowed us to distinguish whether plants can identify neighbours as ‘related’ versus ‘unrelated’, or the degree of relatedness influences the degree of the response. We found that competitive responses of commercial soya bean plants to the identity of neighbours occurred in two traits in response to an unrelated species and in one trait in response to a related ancestral species, and no traits responded at the intra-specific level. These results suggest that phenotypic plasticity to neighbour identity in commercial soya bean may be the result of responses to a gradient of genetic relatedness rather than to a clear-cut signal that would indicate ‘related’ versus ‘unrelated’ individuals.

Commercial soya bean plants showed no differences in any of the measured traits in response to growing with neighbours of the SC or neighbours of DCs, i.e. intraspecific interactions. This lack of response to neighbour identity among cultivars may have resulted from the absence of a recognition system at the intraspecific level or from a lack of sufficient genetic diversity among crop cultivars to trigger a response [39].

In response to wild soya bean, a highly related ancestral species, the commercial soya bean cultivar OAC Wallace displayed identity recognition by increasing biomass allocation to leaves compared with stems and increasing total biomass. There are two possible interpretations for the observed increase in total biomass. One possibility is that the increase in biomass allocation towards leaves in response to neighbours of wild soya bean provided a competitive advantage with a resulting increase in size. A second possibility is that the observed increase in total biomass is the outcome of size-specific competition [40]. Wild soya bean plants are inherently smaller than commercial soya bean plants. If changes in biomass were the result of size-specific competition, then we would expect wild soya bean plants to show a significant reduction in total biomass when growing in the presence of larger commercial soya bean plants. This, however, was not observed, thus suggesting that size-specific competition did not account for the increase in total plant biomass.

In response to the unrelated species *P. vulgaris*, OAC Wallace showed identity recognition by increasing biomass allocation to leaves compared with both stems and roots. Allocation of biomass to resource-acquiring organs such as leaves is expected to increase a plant's ability to acquire resources while reducing resource availability for competitors. These results are consistent with kin selection theory that predicts the development of more competitive phenotypes with less related neighbours. They are also consistent with changes in biomass allocation in response to the identity of neighbours reported earlier in other species such as *Impatiens pallida* [15].

Wild soya bean plants also displayed identity recognition responses. Interestingly, however, in terms of leaf to stem allocation, wild soya bean reduced allocation to leaves in response to more unrelated neighbours. This response was in the opposite direction to that observed in commercial soya bean cultivars. In addition, wild soya bean reduced root to stem allocation. These results suggest an overall resource allocation shift from leaves and roots towards stems in wild soya bean. In the commercial cultivar, however, the shift in allocation occurred from stems and roots into leaves. A possible explanation for this difference in allocation patterns between commercial and wild soya bean may be the result of their different ecologies.

Differential responses to identity recognition based on the ecology of the species are to be expected [12]. Commercial soya bean cultivars are bred for yield and seed quality, grown under intensively managed environments, at optimal densities and in monospecific stands. These conditions create relatively homogeneous environments that have been designed to minimize intra-specific competitive interactions. These same commercial cultivars, however, have the capability to produce more competitive phenotypes (phenotypic plasticity) in the presence of neighbouring weeds, i.e. to interspecific competitive interactions [41]. The results found in this study are consistent with this competitive response to unrelated neighbours. Wild soya bean plants are found growing in natural habitats along river banks, shrubberies and boggy meadows in their centre of origin, China [42]. They display vine-type growth morphology, with slender, weak, branched stems. Contrary to commercial soya bean, wild soya bean plants grow mostly under interspecific conditions with species that display completely different morphologies such as trees, shrubs and grasses. Thus, the observed change in biomass allocation towards stems and away from resource-acquiring organs like leaves and roots may be a competition avoidance strategy that will require stem growth in anticipation of impending competition rather than nutrient foraging. An alternative explanation may be that the observed shift in biomass allocation is due to height differences among plants of wild soya bean and commercial soya bean. If this was the case, however, the shifts in allocation should be accompanied by increases in stem elongation, the most common response to low R:FR, which were not observed in this study.

Whether the differential behaviours observed here by commercial cultivars and the ancestral wild soya bean convey significant fitness benefits is beyond the scope of this study. According to kin selection theory, behaviours that reduce competition towards siblings should provide fitness benefits through increased inclusive fitness, by making resources more available to neighbouring kin, and avoiding the cost of competition. Whether groups of siblings will have higher fitness than groups of unrelated individuals is, however, uncertain. The reason for this is that in addition to possible benefits acquired through kin selection, plants may also experience increased sibling competition in highly related groups. This is known as the ‘elbow room’ or ‘niche partitioning’ hypothesis [43], which predicts that more related individuals, by being more genetically similar, may also be more phenotypically similar and thus less likely to be able to partition a given resource niche. Therefore, final fitness of a group is likely to be determined by the relative strength of these processes that are not mutually exclusive and can co-occur [12].

In conclusion, this study provides evidence that the commercial soya bean cultivar OAC Wallace was capable of identity recognition to, at least, a highly related ancestral species as well as an unrelated species. In addition, this study suggests that identity recognition responses occurred following a gradient of genetic relatedness rather than as a consequence of a clear-cut signal that would indicate ‘related’ versus ‘unrelated’ individuals. Finally, the responses to neighbour identity of the commercial soya bean cultivar OAC Wallace differed from those of wild soya bean, suggesting that they were species-specific and consistent with the ecology of each species. Future studies should consider additional soya bean cultivars as well as other crop species to establish whether identity recognition is a widespread ability in crop plants. Elucidating identity recognition in crops may provide further knowledge on mechanisms of crop competition and the relationship between crop density and yield.

## Data accessibility

Our data are deposited at Dryad: http://dx.doi.org/10.5061/dryad.1k85g [44].
